# Interleukin-1β-stimulated macrophage-derived exosomes improve myocardial injury in sepsis via regulation of mitochondrial homeostasis: experimental research

**DOI:** 10.1097/JS9.0000000000001915

**Published:** 2024-07-05

**Authors:** Chunhua Ma, Zhaocong Yang, Jing Wang, Han She, Lei Tan, Xuming Mo, Tao Li, Liangming Liu

**Affiliations:** a State Key Laboratory of Trauma, Burns and Combined Injury, Shock and Transfusion Research Department of Army Medical Center, Army Medical University, Chongqing; b Children’s Hospital of Nanjing Medical University, Nanjing; c School of Biology and Food Engineering, Institute of Pharmaceutical Pharmacology Research Center, Suzhou University, Suzhou, Anhui, People’s Republic of China

**Keywords:** exosomes, miR-146a, mitochondrial function, sepsis-induced myocardial injury

## Abstract

**Background:**

The purpose of this study was to investigate the effects of interleukin-1β (IL-1β) stimulation on the protection of macrophage-derived exosomes miR-146a (M-IL-exo-146a) on sepsis-induced myocardial injury (SMI) *in vitro* and *in vivo*.

**Materials and methods:**

Macrophage-derived exosomes (M-exo) and IL-1β-stimulated macrophage exosomes (M-IL-exo) were isolated from macrophages of sepsis with or without IL-1β. The expressions of miR-146a in M-exo and M-IL-exo were detected by fluorescence quantitative PCR. Related molecular biology technologies were used to evaluate the role and mechanism of M-exo-146a and M-IL-exo-146a on SMI and the enhancing effect of IL-1β.

**Results:**

Compared with M-exo, the expression of miR-146a in M-IL-exo was significantly increased. M-IL-exo-146a significantly alleviated SMI by decreasing the level of serum myocardial enzymes, serum and myocardial oxidative stress and cytokines, and improved myocardial mitochondrial imbalance. The mechanism responsible for IL-1β enhancing the production of IL-M-exo miR-146a was via JNK-1/2 signal pathway. The mechanism responsible for M-exo-IL-miR-146a protecting SMI was related to miR-146a inhibiting inflammatory response and mitochondrial function via MAPK4/Drp-1 signal pathway.

**Conclusions:**

This study provides a new strategy for the treatment of SMI by delivering M-IL-exo.

## Introduction

Highlights

- Macrophage-derived exosomes miR-146a (M-IL-exo-146a) significantly improved sepsis-induced myocardial injury (SMI) by decreasing the level of serum myocardial enzymes, serum and myocardial oxidative stress and cytokines, and improved myocardial mitochondrial imbalance.
- Interleukin-1β (IL-1β) enhancing the production of IL-M-exo miR-146a was regulated via JNK-1/2 signal pathway.
- M-exo-IL-miR-146a protecting SMI was related to miR-146a inhibiting inflammatory response and mitochondrial function via MAPK4/Drp-1 signal pathway.

Sepsis refers to systemic inflammatory response syndrome (SIRS)^1^ caused by infection, which is a serious life-threatening disease with multiple organ dysfunction. In 2017, the World Health Organization (WHO) reported that there were nearly 30 million patients worldwide every year, and the mortality rate was as high as 50–70%^2,3^. The mortality of sepsis is 30–40% and gradually increasing, and it has become a serious disease threatening human life and health. As the core organ of the body, the heart is also one of the most vulnerable target organs in the course of sepsis^4,5^. Studies showed that sepsis would lead to myocardial inhibition and cardiac systolic and diastolic dysfunction, such as resulting in increased hypertrophy of myocardial structure, decreased left ventricular ejection fraction, etc. Clinical studies reported that about 50% of patients with sepsis have different degrees of myocardial inhibition in the early stage. These patients are prone to hypotension, dizziness, palpitation, and other symptoms. Further aggravation of the condition would lead to cardiac dysfunction and heart failure. The mortality of sepsis patients with myocardial injury is greatly increased, as high as 70–90%. Effective treatment of myocardial injury in sepsis and strengthening myocardial protection in patients with sepsis are not only an important link in the treatment of sepsis but also an important guarantee to improve the survival rate of patients with sepsis^6,7^.

Exosomes are extracellular vesicles with a diameter of 40–200 nm formed by the fusion of multi-vesicles and cell membranes. They are mainly composed of proteins, lipids, and nucleotides, which can promote intercellular communication^8,9^. Previous studies have shown that exogenous endothelial progenitor cells (EPCs) have a protective effect in sepsis, which is characterized by reduced vascular leakage, improved organ function, and improved survival rate. Exosomes have significant immunomodulatory function, low immunogenicity, easy isolation, culture, and rapid expansion *in vitro*, which make exosomes attract much attention in the cell therapy of immune and inflammatory-related diseases. However, recent studies have found that the immune regulation function of exosomes in a resting state is very weak, and the curative effect is not limited. However, after stimulation and activation of inflammatory factors, exosome can give full play to its immune regulation function. Therefore, before treatment with exosomes, pretreatment with appropriate cytokines to enhance its immune regulation function is expected to improve its therapeutic effect and achieve better clinical application. Therefore, the aim of this study was to investigate the effects of interleukin-1β (IL-1β)-stimulated macrophage-derived exosomes miR-146a (M-IL-exo-146a) on sepsis-induced myocardial injury.

## Materials and methods

### Reagent

A small extraction kit for endotoxin-free plasmid was purchased from Qiagen Company of Germany. Biquinoline formic acid (BCA) protein quantitative kit was purchased from Beijing Pulilai Company. Lipopolysaccharide (LPS) and IL-1β were purchased from Sigma Company. Diamino polyethylene glycol was purchased from Yarebio Company. All antibodies were purchased from Cell Signaling Technology Company.

### Animals

C57BL/6J, WT mice (ICR, male, 6 weeks, 16–18 g), *NF-κB1* Knockout mice (C57BL/6J, male, *NF-κB1* ^−/−^, 6 weeks, 16–18 g) were obtained from the Cyagen Biosciences Inc. All animals’ operations were approved by the Research Council and Animal Care and Use Committee of Animal Center of our university and the work has been reported in accordance with the ARRIVE guidelines, Supplemental Digital Content 1, http://links.lww.com/JS9/D32 (Animals in Research: Reporting In Vivo Experiments).

### Sepsis patients

Thirty patients with sepsis in our hospital from January 2020 to December 2020 were included in the study. All patients were diagnosed by clinical examination. The diagnostic criteria were based on the international consensus on the definition of sepsis and septic shock (Sepsis-3) in the third edition. The patients with suspected infection were given a rapid sequential organ failure score (q-SOFA), with changes in consciousness and systolic blood pressure ≤100 mmHg and the respiratory rate ≥22 times/min meets the above two items, it is suspected as sepsis. If the q-SOFA score ≥2 points, it can be diagnosed as sepsis^4^. Among 30 patients with sepsis, there were 17 male patients and 13 female patients; the ages ranged from 51 to 78 years old, with an average age of (65.12±3.43) years old. All patients authorized family members to sign informed consent, and the study was approved by our hospital ethics committee. The above operations were completed by experienced physicians and were double-blind.

#### Case inclusion criteria

(1) 18–76 years old; (2) the survival time in the hospital exceeds 48 h, and the medical records were complete; (3) there was no history of vitamin C use in the recent period of admission (within 2 weeks before admission.

#### Exclusion criteria

(1) Those who died within 24 h after admission to the intensive care unit (ICU) and had drug contraindications for this study; (2) estimated survival time <96 h; (3) non-infection caused systemic inflammatory response syndrome (SIRS) or organ failure (trauma, tumor, etc.); (4) pregnant and lactating women; (5) those who had a long-term history of hormone or immunosuppressive drugs, and those who failed to take drugs continuously during the study period; (6) excluded patients with myocardial infarction or other causes of myocardial damage.

### Blood macrophage extraction from sepsis patient and culture

Macrophages were isolated from the blood of sepsis patients with a commercial kit (Sigma, USA) named M cells. M cells were cultured in Dulbecco’s Modified Eagle’s Medium (DMEM)/Nutrient Mixture F-12 supplemented with 10% fetal bovine serum in a humidified at 37°C under a 5% CO_2_ atmosphere.

### IL-1β-stimulated M cells

M cells were seeded at a 6-well plate at a density of 1×10^5^ cells/well for 24 h. The M cells were divided into the control group and the IL-1β stimulation group. The cells of the IL-1β stimulation group were treated with IL-1β (10 ng/ml) (selection of IL-1β was according to Supplementary Material 1, Supplemental Digital Content 2, http://links.lww.com/JS9/D33) for 24 h, while the control group was challenged with an equal phosphate-buffered saline (PBS). The cells were then collected for standby.

### Extraction of sepsis macrophage-derived exosomes (M-exo)

The ExoQuick Precipitation method was used to extract exosomes, and the specific method was strictly in accordance with the instructions of the kit.

### Exosome’s protein quantification by BCA

1/4 volume of RIPA protein lysate (RIPA protein buffer) was added into macrophage-derived exosomes, mixed evenly, placed in an ice box, and incubated for 30 min until exosomes were completely lysed. Then, the protein contents of M-exo were detected according to BCA kit instructions.

### Identification of M-exo

Western Blot (WB) was used to identify the markers proteins CD63, TSG101, CD9, and CD81 on the surface of exosomes. Transmission electron microscope (TEM) was used to identify the appearance of exosomes; briefly, 15 μl exosomes suspension was dropped onto a 200-mesh copper mesh for sample loading, allowed to stand for 2 min, and the filtrate was sucked dry and stained with 2% uranyl acetate for 15 s, then naturally dried at room temperature, the copper mesh was installed on a 100 KV transmission electron microscope, and the morphology of the exosomes was observed and photographed.

Nanoparticle Tracking Analysis (NTA) was used to identify the size of exosomes. Briefly, the separated exosome samples were appropriately diluted with 1×PBS buffer, and the particle size of the extracellular vesicles was measured using nanoparticle tracking analysis (NTA) (Zeta View system calibrated with 110 nm polystyrene particles, maintained at 23 and 37°C).

### Extraction and analysis of sepsis M-exo RNA

The total RNA of exosomes was extracted using a serum/plasma miRNA extraction kit. The RNA was reversely transcribed into complementary DNA (cDNA) according to the instructions of miScript Ⅱ RT kit, and then the miScript SYBR Green PCR Kit was used to detect RNA. The amplification procedure was 95°C for 15 min; 94°C for 15 s, 55°C for 30 s, 70°C for 30 s, 45 cycles. Five multiple wells were set in all experiments, and the experimental data of quantitative Real-Time PCR (RT-PCR) were 2^−ΔΔ CT^ method (CT is the number of PCR cycles required when the fluorescence reaches the threshold), and the spike in control provided by the kit was used as the internal reference gene. The primer sequences of miR-21-5p, miR-146a, miR-143, miR-147a, and miR-149-5b are shown in Table 1.

**Table 1:** Primer sequences.

miRNAs	Sequences
miR-21-5p	Forward: ACACTCCAGCTGGTAGCTTATCAGACTGA Reverse: CTCAACTGGTGTCGTGAAGTCGGCAATTCAGTTCAGTCAACATC
miR-146a	Forward: ACACTCCAGCTGGGTGAGAACTGAATTCCATGGGTT Reverse: CTCAACTGGTGTCGTGGAGTCGGGCAATTAACCCATGG
miR-143	Forward: ACACTCCAGCTGGGTGAGATGAAGCAC Reverse: CTCAACTGGTGTCGTGGAGTCGGCAATTCAGTTGAGGAGTACA
miR-147a	Forward: ACACTCCAGCTGGGGGTGTGGAAATGC Reverse: CTCAACTGGTGTCGTGGAGAGTCGGCAATT CAGTTGAGGCAGAAGC
miR-149-5p	Forward: ACACTCCAGCTGGGGTCTGGCTCCGTTCTTC Reverse: CTCAACTGGTGTCCGTGGAGTCGGCAATTTTCAGGGGAGTGA
U6	Forward: CTCGCTTCGGCAGCACA Reverse: AACGCTTCACGAATTTGCGT
MAPK4-mut	Forward: CTAGCTAGCGCAGACTGGTTTGGGTAAGTCATCTAAGGGAGCTGGT Reverse: GCTCTAACCACCAGCTCCCTTAGATGACTTACCCAAACCAGTCTGC
MAPK4-wt	Forward: GCTCTAGAACTGCATTGCCAGTGTCTAC Reverse: CCGCTCGAGTAGACACTGGCAATGCAGT
ERK-1	Forward: TCTTGCCCTCTTGGTGGT Reverse: AGTCGGCTCCAAATTCCT
CREB	Forward: TCTACAGGCTGCCCAAAA Reverse: CTCAAAAGTCAACCTTTGA
MIFT	Forward: GTCTGACTCACAGGCACTC Reverse: GTCTGACTCAGGCACTC
Rab27α	Forward: TCACGACATCGGCATTG Reverse: GCTCCTGGCTTCTTCCTCT
ML-PH	Forward: GAAGGCACCACCAGGACT Reverse: CAGACAAATCACTCCACCAA

### Fluorescent localization of exosomes *in vitro*

Exosomes were added to 0.3 ml diluent, then 4 μl of PKH67 staining solution was added and incubated at 37°C for 5 min, and unbound excess dye was removed by PBS three times, and then 2 ml of 2% bovine serum albumin (BSA) was added to terminate the reaction for confocal photography.

### Establishment of SMI mice model

Mice were divided into the following groups (*N*=10/group): Ctrl, WT+LPS SEP, WT+LPS+M-exo-146a (M-146a, 30 µg/kg), WT+LPS+M-IL-exo-146a (M-IL-146a, 30 µg/kg), *NF-κB1* ^−/−^ +LPS (KO), *NF-κB1* ^−/−^+LPS+IL-M-exo-146a KO-M-IL-146a, 30 µg/kg), *NF-κB1* ^−/−^+LPS+M-exo-146a (KO-M-146a, 30 µg/kg). The SMI model of mice was established by intraperitoneal (i.p.) injection of lipopolysaccharide (LPS 10 mg/kg). The WT mice (Ctrl) were given the same amount of saline in the same way. After 24 h of intervention, M-exo-146a and M-IL-exo-146a (30 mg/kg) (tail vein injection) (the dose of exo was selected according to the previous report and adjusted appropriately^10^) were given to mice. Mice were treated with an i.p. injection of pentobarbital sodium (50 mg/kg) to induce general anesthesia before an operation, then the serum and heart tissue of the same mice were collected for subsequent testing. Due to the presence of mouse mortality rate in SMI, the final biochemical indicators were tested using six valid samples per group for statistical purposes. Western blotting, cardiac immunohistochemistry, and cardiac pathology evaluation were evaluated using three valid samples per group for statistical purposes. The above operations were completed by experienced experimental technicians and were double-blind.

### In-vitro model establishment

H9c2 cells were divided into following groups (*N*=6/group): control, LPS (50 µM), LPS+M-exo-146a (M-146a, 10 µg/ml), LPS+M-IL-exo-146a (M-IL-146a, 10 µg/ml), LPS+*NF-κB1* siRNA (N-siRNA), LPS+*NF-κB1* siRNA+M-exo-146a (N-siRNA-M-146a, 10 µg/ml), LPS+*NF-κB1* siRNA+M-IL-exo-146a (N-siRNA-M-IL-146a, 10 µg/ml). The cells were treated with M-exo-146a (10 µg/ml) and M-IL-exo-146a (10 µg/ml) (the dose of exo was selected according to the previous report and adjusted appropriately^11^) for 24 h, and 50 µM LPS were co-incubated for 24 h, while the control group was challenged with an equal PBS. Cell supernatant and cells were collected for subsequent detection. The final biochemical indicators were tested using six valid samples per group for statistical purposes. Western blotting, cardiac immunohistochemistry, and cardiac pathology evaluation were evaluated using three valid samples per group for statistical purposes. The above operations were completed by experienced experimental technicians and were double-blind.

### Cell transfection

The H9c2 cells were inoculated in a 6-well plate with 5×10^4^ cells per well and cultured in DMEM containing 10% fetal bovine serum in an incubator based on 37°C and 5% CO_2_. When the cells grew to 50–70% fusion degree, various shRNA were transfected with Translipid HL Transfection Reagent with a final concentration of 80 nmol/l according to the instructions of Translipid HL Transfection Reagent. Before transfection, the cells were cultured in serum-free DMEM medium 2 ml for 2 h, and after transfection for 6 h, the conventional DMEM medium containing 10% fetal bovine serum was changed for further culture. Six hours after transfection, the expression of green fluorescent protein in H9c2 cells was observed under the fluorescence microscope. The transfection efficiency was evaluated by counting the percentage of transfected cells in the total number of cells. WB was used to evaluate the success of cell transfection. The small interfering RNA (siRNA) targeted for *NF-κB1* siRNA (antisense, 5′ CUUUAACGUCGGCUUGGGCUC -3′, and sense, 5′ GCCCAAGCCGACGUUAAAGUA -3′, both synthesized by Sigma-Aldrich Company, was designed on the basis of the Thomas Tuschl protocol. Lyophilized single-stranded RNA oligonucleotides were resuspended in sterile RNase-free water (100 μM), denatured (heated at 95°C for 5 min), aligned, and slowly annealed with decreasing temperature, resulting in the formation of double-stranded siRNA at 50 μM.

### Measurement of myocardial enzymes (CK), myocardial isozymes (CK-MB)

At the end of the experiment, commercial kits detected the levels of CK and CK-MB in serum, heart, and H9c2 cells supernatant. The operation process were strictly followed the instructions of the kits.

### Measurement of reactive oxygen species (ROS) in heart and H9c2 cell mitochondria

Ten milligrams of heart tissue were added with 1:10 normal saline and centrifuged at 12 000 r/min for 10 min, and the single-cell suspension was collected for detecting ROS by ROS detection kit (Beyotime). H9c2 cells were seeded at a 6-well plate at a density of 1×10^6^ cells/well for 24 h. H9c2 cells were collected and mitochondrion were extracted with a mitochondrial extraction kit. Fluorescent probe (1:1000) was used. The samples (cardiac single-cell suspension and H9c2 mitochondrial) were carefully cleaned, centrifuged, and cultured in the incubator for 30 min. The fluorescence of dichlorofluorescein (DCF) was then measured using a laser scanning microscope (Nikon). The average fluorescence intensity was quantitatively analyzed using ImageJ software (National Institutes of Health).

### Biochemical measurement

The levels of superoxide dismutase (SOD), malondialdehyde (MDA), and glutathione peroxidase (GSH-Px) were detected by commercial kit, and the operation method was carried out according to the kits instructions.

### Cytokine measurement

The concentrations of IL-6, IL-1β, and tumor necrosis factor-α (TNF-α) in serum, heart tissues, and cell supernatant were analyzed by enzyme-linked immunosorbent assay (ELISA) kits according to the manufacturer’s instructions.

### Hematoxylin–eosin (HE) staining

The heart was fixed in 4% formalin solution, embedded in paraffin, cut into 4 μm thick sections, and placed on a glass slide for HE staining.

### Color Doppler ultrasound examination of mice heart

Mice were anesthetized with isoflurane, VEVO 2100 ultrasonic imager equipped with a 13–24 MHz sensor was used for detection, and two-dimensional short-axis M-mode echocardiography was performed at the middle level of papillary muscle, the above operations were completed by experienced experimental technicians and were double-blind.

### Exosome myocardial localization

Sixty microliters of fluorescent agent-labeled exosome (DIR-exo) were injected into the septic mice via tail vein. Immediately and 2 h after injection, the septic mice were put into a small animal living imager (IVIS Spectrum) for back and abdomen living imaging; the above operations were completed by experienced experimental technicians and were double-blind.

### Mitochondrial localization of exosome in H9c2 cells

H9c2 cells were seeded at a 6-well plate at a density of 1×10^6^ cells/well for 24 h. After that, the cells were incubated with PKH67-exosome (10 µg/ml) for 24 h, and then the cells were observed by laser confocal microscope.

### Mitochondrial respiration function

The oxygen consumption rate (OCR) was measured using a 24-well XFe plate (Seahorse, Agilent Cell Analysis technology, USA). H9c2 cells were seeded at a density of 1×10^4^ per well, and put the cell plate in the super-clean bench for 1 h to make the cells settle naturally. Then, the cell plate was put back into the cell incubator overnight to make the cells adhere to the wall. When the cell confluence reached 70–80%, exo (10 µg/ml) was added to treat cells for 12, 24, and 48 h. Before detection, H9c2 cells were added LPS. Then, the basic assay medium containing 2.5 μM glucose and 2 mM glutamine was used to culture cells for 50 min. Then 2 μM oligomycin, 1 μM FCCP, and 0.5 μM rotenone/antimycin A were performed sequentially. The OCR was measured by an extracellular flux analyzer under the mitochondrial stress test condition.

### TEM observation of myocardial tissue in sepsis

Fresh heart tissues were cut into squares with no more than 1 mm×1 mm×1 mm, and then were quickly put into fixing solution at 4°C for 2–4 h, and then at room temperature (20°C) for 1 h. 100% acetone was used to dehydrate and then embedded in 812 solutions (epoxy resin), dried at 60°C for 48 h, cut as 60–80 nm ultra-thin slices, dyed, and finally placed for TEM observation.

### Verification of the mechanism of miRNA146a regulating mitochondrial dysfunction

#### miRNA146a targeted *MAPK4* detection

*Construction of prim-GL0-MAPK4-3′ double fluorescent report vector*: Total RNA was extracted from H9c2 cells and amplified by QPCR using reverse transcription cDNA as a template. The purified product of PCR amplification and prim GL0 vector was digested with *NheI* and *XbαI*, respectively, at 37°C for 3 h. The product was linked to DH5α at 4°C. Plaque bacteria were picked up, and the plasmid was extracted and sequenced after shaking.

*H9c2 cell transfection*: H9c2 cells were inoculated into six-well plates (1×10^6^). When the cell density reached 75%, the cells were transfected with miRNA146a mimic, inhibitor and negative control (NC), and each group was repeated for three times.

*Detection of the targeting relationship between miRNA146a and MAPK4 by double luciferase reporter gene assay*: 100 μl of fish phospholipase B was added to cells/each well, the suspension was added to 96 well plates (50 μl/per well), then 100 μl LAR-II was added, the fluorescein reaction intensity value was recorded at the same time, then 100 μl of reaction termination solution was added, and finally the fluorescein reaction intensity value of sea kidney fluorescein was recorded. The results were expressed by firefly fluorescein reaction intensity value/firefly fluorescein reaction intensity value.

*Detection of related gene and protein expression by PCR and WB*: Total RNA of transfected cells was extracted by Trioal. After the concentration was determined, reverse transcription was performed using a reverse transcription kit, and then fluorescent PCR was performed using a AeCQ PCR SYBR Green Master Mix kit.

The transfected cells were obtained, the loading concentration was calculated, SDS-PEG gel electrophoresis was performed, the primary antibodies were incubated at 37°C, then the primary antibodies were washed, secondary antibodies were incubated, ELC luminescent solution was added for development and obtained pictures.

#### In-vitro phosphorylation of Drp-1 by MAPK4

At 30 μl reaction mixture [40 mm Tris-HCl, pH=7.5, 2 mm dithiothreitol, 10 mM MgCl_2_, and 100 μl adenosine triphosphate (ATP)], 500 ng of recombinant human Drp-11 (GST-labeled protein, Abnova, Taiwan) was incubated with 250 ng of recombinant human MAPK4 (Prospect). After incubation at 30°C for 30 min, the reaction was stopped by boiling in the sample loading buffer of sodium dodecyl sulfate–polyacrylamide gel electrophoresis (SDS–PAGE). Anti-serine threonine phosphorylation antibody was used to detect the phosphorylation of Drp-1.

### In-vitro validation of the mechanism of IL-β on miRNA146a

#### Effect of IKK-2 and JNK-1/2 inhibitors on the effect of IL-β on miRNA146a

The M cells were inoculated in a 6-well plate with 5×10^4^ cells per well and cultured in DMEM containing 10% fetal bovine serum in an incubator based on 37°C and 5% CO_2_. The M cells were cultured for an additional 1 h in the presence or absence of the indicated concentrations of TPCA-1 (an IKK-2 inhibitor) and SP600125 (a JNK-1/2 inhibitor) and then stimulated with 50 µM of IL-1β for 0, 10, 30, 60, and 120 min. The rest of the cells were cultured for 24 h, the M cells were collected, and the level of miRNA146a was detected by QPCR.

#### The effects of IL-β on the uptake of exo-miRNA146 by H9c2 cells

The experiment was carried out with the Transwell model of M cells in the upper chamber and H9c2 cells in the lower chamber. The experiment was divided into the control group (there was no IL-1β in the upper chamber) and the treatment group (upper chamber including 50 μM IL-1β). After 4, 8, and 12 h, the corresponding Transwell chamber was taken out, H9c2 cells in the lower chamber were collected, PKH67 staining was performed, and then DAPI was added to incubate for 15 min. The uptake of exo-miRNA146a by H9c2 cells in the lower chamber was observed. At the same time, miRNA146a was detected by QPCR.

### Immunohistochemistry

Heart tissue slices were baked at 60°C for 1 h, then paraffin was removed by xylene, dehydrated by gradient ethanol, and heated by sodium citrate buffer for antigen repair. After natural cooling to room temperature, it was cultured with 3% hydrogen peroxide for 10 min. Each section was sealed with 3% BSA at room temperature for 1 h. After removing the blocking solution, the section was incubated with the primary antibody at 4°C overnight, the secondary antibody was incubated for 10 min, and PBS was washed three times. The third antibody was incubated for 10 min and washed with PBS for 3 min each time. Samples were stained with diaminobenzidine (DAB) and hematoxylin, dehydrated by gradient ethanol and xylene, dried, and sealed with neutral resin, and the expression of related proteins in lung tissue was observed under a light microscope.

### WB analysis

Total protein was extracted from the lysate of heart tissue and H9c2 cells, and the protein content was determined by the BCA method. SDS–PAGE electrophoresis was performed on protein samples and transferred to the polyvinylidene fluoride (PVDF) membrane. The 5% skimmed milk powder was used to seal at room temperature for 2 h, and incubated with related equal primary antibodies, respectively. The membrane was rinsed with tris-buffered saline with Tween-20 (TBST) and then reacted with horseradish peroxidase (HRP) coupled secondary antibody. The membrane was rinsed with TBST and then developed with enhanced chemiluminescence luminescent reagent (ECL). The optical density of the main band was measured by gray-scale imaging software (UVP, UK) to calculate the expression level of the above proteins in lung tissue.

### Immunofluorescence

The levels of LC-32 in H9c2 cells were evaluated by immunofluorescence. Briefly, cultured H9c2 were washed twice with PBS, fixed with 4% paraformaldehyde (PFA) for 30 min, and then permeabilized with 0.5% Triton X-100 in PBS for 5 min, blocked with 5% BSA for 1 h. The cells were incubated with the primary antibody LC-32 (1:500) overnight at 4°C, washed three times with PBS, and incubated with goat anti-rabbit IgG (H+L) secondary antibody, Alexa Fluor 488 conjugate (1:500) for 1 h. After washing with PBS three times, the DAPI was done at room temperature for 5 min. Fluorescence images were taken with fluorescence microscopy.

### Immunoprecipitation (IP)

According to the instructions of the immunoprecipitation kit, 20 μg Amino link plus resin and Pierre Control Agarose Resin (the negative control, which does not react with antibody to remove non-specifically bound protein) were incubated with 4 μg MAPK4 rabbit monoclonal antibody for 90 min. A 500 μl (about 1 mg) of protein sample was added overnight at 4°C, it was washed with IP lysis/wash buffer for four times, then it was washed with conditions buffer for one time, and it eluted MAPK4 protein complex with solution buffer. The above sample was used for 10% SDS–PAGE electrophoresis and WB. 1:1000 diluted rabbit anti-human LC-32 primary antibody and 1:1000 diluted rabbit anti-human Drp-1 primary antibodies were added, respectively, overnight at 4°C, after TBST washing three times, the corresponding HRP-labeled secondary antibody was added, and incubated at room temperature for 1 h. After TBST washing three times, ECL Chemical solutions was used to display stripes.

### Statistical analysis

All data were expressed as mean±standard deviation (SD) and analyzed by one-way analysis of variance (ANOVA) followed by Tukey multiple comparison test using GraphPad Prism 5 (GraphPad Software, USA). A value of *P*<*0.05* was considered statistically significant.

## Results

### Extraction and identification of primary macrophages from sepsis patients

**Figure 1:**
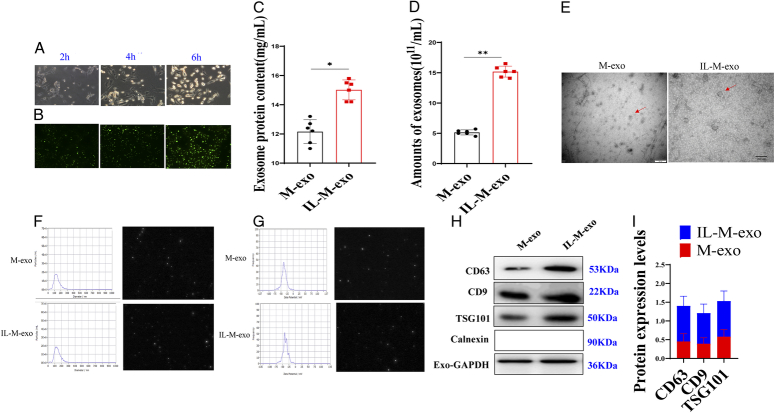
Extraction and identification of primary macrophages from sepsis. (A) Observation of blood primary macrophages by inverted microscope. (B) Macrophage CD63 staining. (C) Quantitative detection results of protein in exosomes. (D) Quantity of exosomes. (E) M-exo and M-IL-exo were observed by transmission electron microscope. (F) Particle size of M-exo and M-IL-exo. (G) Potential of M-exo and M-IL-exo. (H) The results of Western Blot of exosome marker proteins of M-exo and IL-M-exo. (I) Quantity of exosome marker proteins of M-exo and IL-M-exo.

The number of macrophages (M) in l ml of peripheral blood from sepsis patients was measured by cell counting. The number of M cells was (3.17±0.13)×10^6^/ml. They appeared not even in size and shape (Fig. 1A). Immunocytochemical detection showed that the specific surface marker CD68 on M cells from sepsis patients was positive (Fig. 1B).

### Protein content and morphology of exo

The average protein concentrations in M-exo and IL-M-exo were 12.58 mg/ml and 15.63 mg/ml, respectively (Fig. 1C). Meanwhile, the amounts of eco were 5.26×10^11^/ml and 15.32×10^11^/ml, respectively (Fig. 1D).

TEM observation showed that the exosomes extracted from M-exo and IL-M-exo were all round and round-like vesicles, the membranous structures were clearly visible, and all exosomes were distributed singly or clustered (Fig. 1E).

### NTA test results

Hundred microliters of exosomes extracted from M cells and IL-M cells were added into PBS diluted to 5 ml and detected by the nanoparticle tracking analyzer. The results showed that the viscosities of the dispersion media of M-exo and M-IL-exo were 0.894 mPa s and 0.895 mPa s, respectively. The transmission amounts of M-exo and M-IL-exo were 29 782 and 31 347 particles, respectively. The detected particle count rates were 1125 (kCPS) and 1649 (kCPS). The MODE curve was linear and smooth, indicating that the purity of exo was high. The peak particle sizes were 124.8 nm and 113.6 nm, respectively (Fig. 1F, G).

### Exosome markers CD63, CD9, Tsg101, and calnexin protein

WB was used to detect the exo marker proteins CD63, CD9, Tsg101, and calnexin in M-exo and M-IL-exo. Results showed that CD63, CD9, and Tsg101 had the expression, but calnexin had no expression, which indicated that the extraction was better, the exosomes were not interfered with by other proteins, and the purification was good (Fig. 1H, I).

### Cardiac tissue targeting

**Figure 2:**
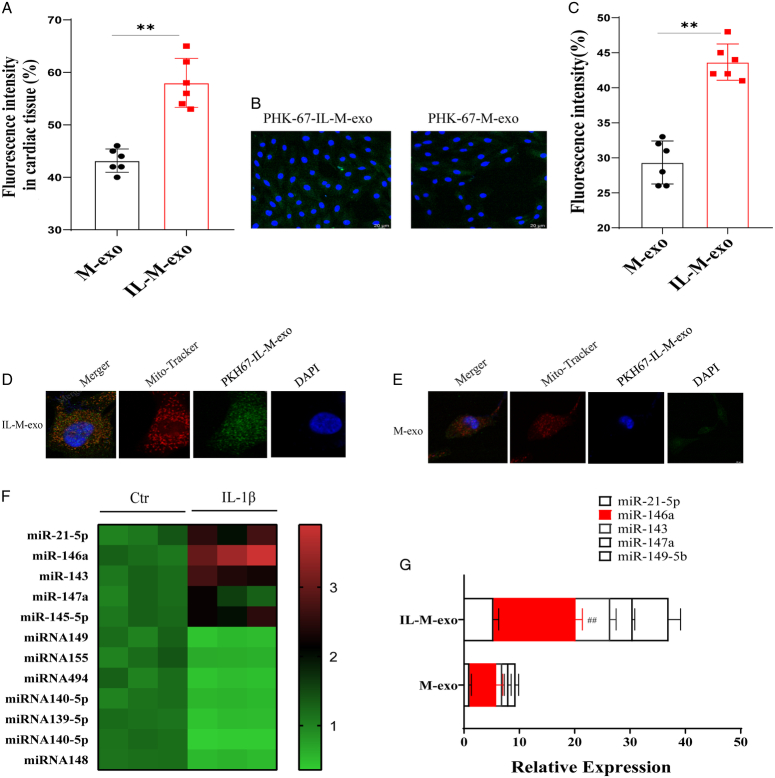
Localization of M-exo and M-IL-exo target tissues and target cells and expression level of miR-146a. (A) Fluorescence intensity quantification of M-exo and M-IL-exo at 2 h in mice. (B) Distribution of M-exo and M-I-exo at 2 h in H9c2 cells. (C) Fluorescence intensity quantification of M-exo and M-IL-exo at 2 h in H9c2 cells. (D) Distribution of M-IL-exo at 2 h in H9c2 mitochondrion. (E) Distribution of M-exo at 2 h in H9c2 mitochondrion. (F)Expression levels of miRNA146a in M-exo and M-IL-exo in Hotspot map. (G) Expression levels of miRNA146a, miRNA21-5p, miRNA143, miRNA147a, and miRNA149-5p in M-exo and M-IL-exo.

The in-vivo imaging results of small animals showed that both DiR-M-exo and DiR-M-IL-exo could reach heart tissue 2 h after administration via the tail vein, the fluorescence intensity of DiR-IL-M-exo was higher than that of DiR-M-exo (Fig. 2A, B).

### The mitochondrial localization of M-exo and M-IL-exo in H9C2 cells

Whether M-exo and M-IL-exo can reach the mitochondria to exert its effect is the important issue, so the location of M-exo and M-IL-exo in H9c2 cells was detected, as shown in Figure 2D–F, PHK-67-M-exo, and PHK-67-IL-M-exo collocated with mitochondria (Red MitoTracker) in H9c2 cells. The fluorescence intensity of PHK-67-M-IL-exo was higher than that of DiR-M-exo (Fig. 2C–F).

### Expression levels of miR-146a in M-exo and M-IL-exo

Through RNA chip technology (with 50 RNAs), 12 differential RNAs were found between M-exo and M-IL-exo (Fig. 2H). In this study, we investigated several miRNAs with important functions (miR-21-5p, miR-146a, miR-143, miR-147a, and miR-149-5b). The expression levels of these miRNAs in M-exo and M-IL-exo were detected by quantitative real-time PCR (QPCR). The results showed that the expression level of miRNA146a was the highest in M-exo and M-IL-exo, and the level of miRNA146a was significantly higher than that in M-IL-exo than M-exo (Fig. 2I).

### M-IL-exo-146a alleviated the percent survival, ROS, CK, and CK-MB in sepsis mice

**Figure 3:**
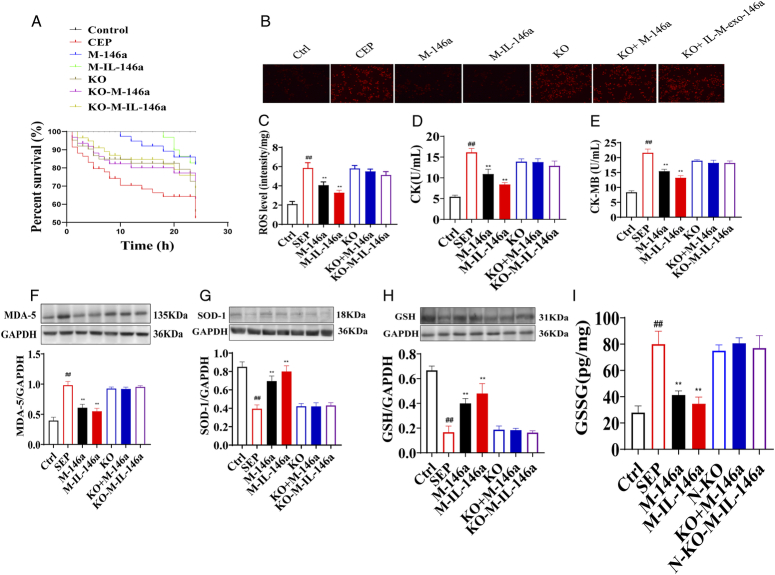
The effects of M-IL-exo-146 on percent survival, ROS, CK, CK-MB, and oxidative stress (*n*=6). (A) Percent survival. (B) ROS fluorescence probe detection. (C) ROS fluorescence intensity quantification. (D) CK in serum. (E) CK-MB in serum. (F) MDA-5 in heart. (G) SOD in heart. (H) GSH-px in heart. (I) GSSG in heart. Ctrl: WT mice (control); SEP: LPS; M-146a: LPS+M-exo-146a (30 µg/kg); M-IL-146a: LPS+M-IL-exo-146a (30 µg/kg); KO: *NF-κB1* ^−/−^+LPS, KO+M-146a: *NF-κB1* ^−/−^+LPS+M-exo-146a (30 µg/kg); KO+M-IL-146a: *NF-κB1* ^−/−^+LPS+M-IL-exo-146a (30 µg/kg). All the data were presented as mean±SD. Compared with the control group: ^#^ *P*<0.05 and ^##^ *P*<0.01. Compared with the SEP group: ^*^ *P*<0.05 and ^**^ *P*<0.01.

Mouse percent survival is a golden indicator for evaluating the treatment of sepsis. All mice in the control group survived. Compared with the control group, the percent survival of mice in the group significantly decreased. Compared with the LPS group, the percent survival of the M-exo-146a and M-IL-exo-146a groups showed an effective reversal, and M-IL-exo-146a has more effect on percent survival, *NF-κB1* ^−/−^ canceled the effects of M-exo-146a and IL-M-exo-146a on percent survival [percent survival: *F*(6, 126)=23.58, *P*<0.01]. The levels of ROS (Fig. 3A, B), CK (Fig. 3C), and CK-MB (Fig. 3D) in heart tissues were significantly increased in the sepsis group as compared with the control group. Meanwhile, M-exo-146a and M-IL-exo-146a significantly decreased the levels of ROS, CK, and CK-MB in heart tissues in sepsis mice, M-IL-exo-146a had a better effect in reducing the level of ROS, CK, and CK-MB. While, *NF-κB1* ^−/−^ canceled the effects of M-exo-146a and IL-M-exo-146a on ROS, CK, and CK-MB [ROS: *F*(6, 35)=101.5, *P*<0.01; CK: *F*(6, 35)=113.6, *P*<0.01; CK-MB: *F*(6, 35)=190.1, *P*<0.01].

### M-IL-exo-146a alleviated oxidative stress, heart histopathology injury and inflammation, and improved the cardiac function and mitochondrial function in sepsis mice

The levels of MDA-5 and oxidized glutathione (GSSG) in heart tissues in sepsis mice were significantly increased, while SOD and GSH-px were significantly decreased as compared to the control group. M-exo-146a and M-IL-exo-146a significantly decreased the MDA and GSSG and increased the SOD and GSH-px in the heart tissues of sepsis mice; M-IL-exo-146a had a stronger effect. *NF-κB1* ^−/−^ canceled the effects of M-exo-146a and M-IL-exo-146a on the oxidative parameters (Fig. 3E–H) [MDA: *F*(6, 14)=82.82, *P*<0.01; SOD: *F*(6, 35)=112.2, *P*<0.01; GSH-px: *F*(6, 14)=62.92, *P*<0.01; GSSG: *F*(6, 14)=41.33, *P*<0.01].

**Figure 4:**
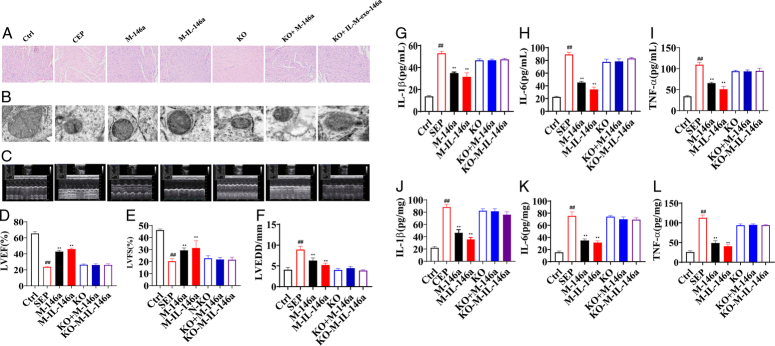
The effects of M-IL-exo-146 on heart histopathology and oxidative stress and inflammation in sepsis mice. (A) Representative heart tissue sections photomicrographs for hematoxylin–eosin (HE) staining (*n*=3). Original magnification: 200, scale bar: 50 μm. (B) Cardiac electron microscope results. (C) Result of color sonography. (D) The level of LEVF (%). (E) The level of LVFS (%). (F) The level of LVEDD. (G) Serum level of IL-1β. (H) Serum level of IL-16. (I) Serum level of TNF-α. (J) Heart level of IL-1β. (K) Heart level of IL-6. (L) Heart level of TNF-α. Ctrl: WT mice (control); SEP: LPS; M-146a: LPS+M-exo-146a (30 µg/kg); M-IL-146a: LPS+M-IL-exo-146a (30 µg/kg); KO: *NF-κB1* ^−/−^+LPS, KO+M-146a: *NF-κB1* ^−/−^+LPS+M-exo-146a (30 µg/kg); KO+M-IL-146a: *NF-κB1* ^−/−^+LPS+M-IL-exo-146a (30 µg/kg). All the data was presented as mean±SD. Compared with control group: ^#^ *P*<0.05 and ^##^ *P*<0.01. Compared with SEP group: ^*^ *P*<0.05 and ^**^ *P*<0.01.

Heart pathological results showed that the myocardial fibers in sepsis mice were arranged loosely, and there was a large number of inflammatory cells infiltration. M-exo-146a and M-IL-exo-146a alleviated the damage to the heart, and IL-M-exo-146a had a better effect. *NF-κB1* ^−/−^ canceled the effects of M-exo-146a and IL-M-exo-146a (Fig.4A).

The TEM results showed myocardial fiber arrangement disorder, mitochondrial swelling, and crista reduction in the sepsis group. As compared with sepsis mice, the disordered arrangement of myocardial fibers, the swelling of mitochondria, and the reduction of cristae were improved in the M-exo-146a group and the IL-M-exo-146a group. The IL-M-exo-146a group had a better effect. *NF-κB1* ^−/−^ canceled the effects of M-exo-146a and IL-M-exo-146a (Fig. 4B). For the morphology change of myocardial mitochondria, in the control group, the mitochondria were basically regular, the boundary was clear, the matrix was uniform, the mitochondrial ridge was dense, and there was no obvious swelling and vacuolar degeneration. After LPS stimulation, the size of normal mitochondria was decreased, and the number of damaged mitochondria was increased. However, M-exo-146a and M-IL-exo-146a significantly restored the above changes in heart mitochondria. M-IL-exo-146a had better effect than M-exo-146a. *NF-κB1* siRNA canceled the effects of M-exo-146a and M-IL-exo-146a (Fig. 4B).

Cardiac function is an important index in evaluating myocardial disease in sepsis. Compared with the control group, the left ventricular ejection fractions (LVEF) and shortening rate of the left ventricular short axis (LVFS) were significantly reduced, and left ventricular end-diastolic dimension (LVEDD) and left ventricular end-systolic diameter (LVESD) were significantly increased in the sepsis group. M-exo-146a and M-IL-exo-146a significantly increased LVEF and LVFS and decreased LVEDD and LVESD of sepsis mice. *NF-κB1* ^−/−^ canceled the effects of M-exo-146a and M-IL-exo-146a (Fig. 4C–F) [LVEF: *F*(6, 35)=619.4, *P*<0.01; LVSF: *F*(6, 35)=63.16, *P*<0.01; LVEDD: *F*(6, 35)=76.77, *P*<0.01].

In order to evaluate inflammatory reaction, cytokines (TNF-α, IL-1β and IL-6) in serum and heart tissues were detected. As expected, the levels of TNF-α, IL-1β, and IL-6 in serum (Fig. 4G–I) and heart tissues (Fig. 4J–L) were increased in sepsis mice as compared with the control group. M-exo-146a and M-IL-exo-146a significantly decreased the levels of TNF-α, IL-1β, and IL-6 in serum and heart tissues. IL-M-exo-146a had stronger effect on TNF-α, IL-1β, and IL-6. *NF-κB1* ^−/−^ canceled the effects of M-exo-146a and M-IL-exo-146a in serum [TNF-α: *F*(6, 35)=215.6, *P*<0.01; IL-1β: *F*(6, 35)=355.8, *P*<0.01; IL-6: *F*(6, 35)=451.7, *P*<0.01] and heart tissues [TNF-α: *F*(6, 35)=373.1, *P*<0.01; IL-1β: *F*(6, 35)=264.0, *P*<0.01; IL-6: *F*(6, 35)=288.9, *P*<0.01].

### The effect of M-IL-exo-146a on protein expression of mitochondrial respiratory complex (CIII-core2, CII-30, and CIV-II) in myocardial tissue in mice

**Figure 5:**
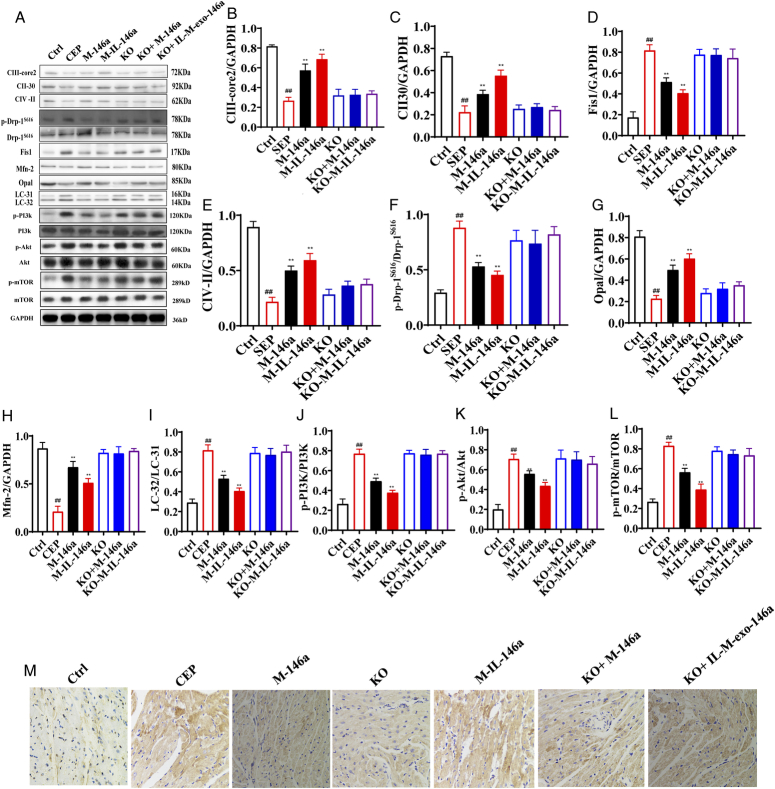
The effects of M-IL-exo-146 on mitochondrial respiration, fusion division, autophagy-related proteins protein in the heart. (A) The levels of CIII-core2, CII-30, and CIV-II, p-Drp-1^S616^, Fis1, Mfn-2, Opal, LC-32/LC-31, p-PI3K, p-Akt. and p-mTOR were detected via Western Blot. (B) The quantification levels of CIII-core2. (C) The quantification level of CII-30. (D) The quantification level of CIV-II. (E) The quantification level of p-Drp-1^S616^. (F) The quantification level of Fis1. (G) The quantification level of Mfn-2. (H) The quantification level of Opal. (I) The quantification level of LC-32/LC-31. (J) The quantification level of p-PI3K. (K) The quantification level of p-Akt. (L) The quantification level of p-mTOR. (M) Heart immunohistochemistry of LC-32 (*n*=3). Original magnification: 200, scale bar: 20 μm. Ctrl: WT mice (control); SEP: LPS; M-146a: LPS+M-exo-146a (30 µg/kg); M-IL-146a: LPS+M-IL-exo-146a (30 µg/kg); KO: *NF-κB1* ^−/−^+LPS, KO+M-146a: *NF-κB1* ^−/−^+LPS+M-exo-146a (30 µg/kg); KO+M-IL-146a: *NF-κB1* ^−/−^+LPS+M-IL-exo-146a (30 µg/kg). All the data were presented as mean±SD. Compared with control group: ^#^ *P*<0.05 and ^##^ *P*<0.01. Compared with SEP group: ^*^ *P*<0.05 and ^**^ *P*<0.01.

As shown in Figure 5A–L, compared with the control group, the levels of CIII-core2, CII-30 and CIV-II were decreased in the sepsis group. M-exo-146a and M-IL-exo-146a significantly increased the levels of CIII-core2, CII-30, and CIV-II in heart tissues. M-IL-exo-146a had better effect on CIII-core2, CII-30, and CIV-II. *NF-κB1* ^−/−^ canceled the effects of M-exo-146a and M-IL-exo-146a [CIII-core2: *F*(6, 14)=60.82, *P*<0.01; CII-30: *F*(6, 14)=68.96, *P*<0.01; CIV-II: *F*(6, 14)=71.33, *P*<0.01].

### The effect of M-IL-exo-146a on myocardial mitochondrial division and fusion in mice

As shown in Figure 5A–L, compared with the control group, the levels of p-Drp-1^S616^ and Fis1 were significantly increased, and Mfn-2 and Opal were significantly decreased in sepsis mice. M-exo-146a and M-IL-exo-146a significantly markedly increased the levels of Mfn-2 and Opal and decreased p-Drp-1^S616^ and Fis1 in the heart tissues of sepsis mice. M-IL-exo-146a had better effect. *NF-κB1* ^−/−^ canceled the effects of M-exo-146a and IL-M-exo-146a [Mfn-2: *F*(6, 14)=62.40, *P*<0.01; Opal: *F*(6, 14)=65.73, *P*<0.01; p-Drp-1^S616^/p-Drp-1^S616^: *F*(6, 14)=28.63, *P*<0.01; Fis1: *F*(6, 14)=55.74, *P*<0.01].

### The effects of M-IL-exo-146a on myocardial mitochondrial autophagy in mice

As shown in Figure 5A–L, compared with the control group, the levels of LC-32/LC-31, p-PI3K, p-Akt, and p-mTOR were increased in sepsis mice. M-exo-146a and M-IL-exo-146a significantly decreased the levels of LC-32/LC-31, p-PI3K, p-Akt, and p-mTOR in heart tissues. IL-M-exo-146a had better effect. *NF-κB1* ^−/−^ canceled the effects of M-exo-146a and IL-M-exo-146a. The immunohistochemical observation of LC-32 obtained similar results to above experiments (Fig. 5M) [LC-32/LC-31: *F*(6, 14)=54.92, *P*<0.01; p-PI3K/PI3K: *F*(6, 14)=92.21, *P*<0.01; p-Akt/Akt: *F*(6, 14)=29.68, *P*<0.01; p-mTOR/mTOR: *F*(6, 14)=64.62, *P*<0.01].

### The effects of M-IL-exo-146a on inflammatory reaction, ROS, CK, CK-MB, and oxidative stress LPS-induced H9c2 cell

**Figure 6:**
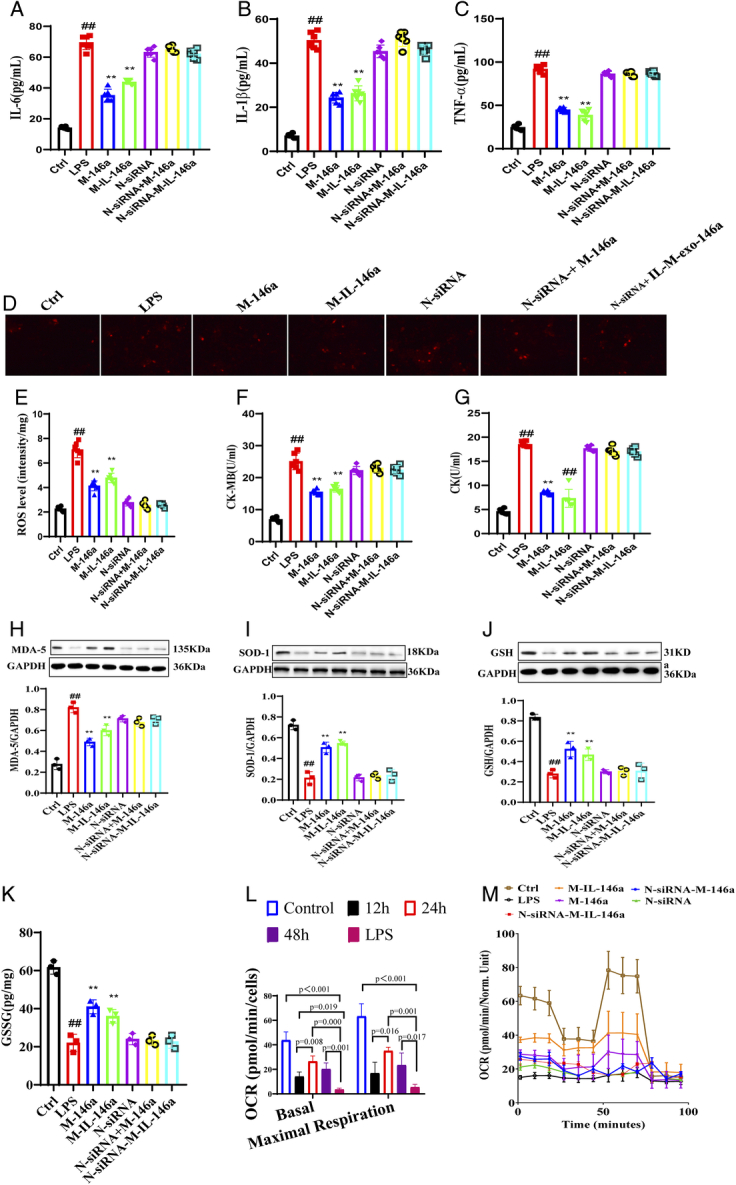
The effects of M-IL-exo-146 on inflammatory reaction, CK, CK-MB, and ROS in H9c2 cells. (A) The level of IL-6 in H9c2 cells (*n*=6). (B) The level of IL-1β in H9c2 cells. (C) The level of TNF-α. (D) ROS fluorescence probe detection in H9c2 cell. (E) ROS fluorescence intensity quantification. (F) The level of CK in H9c2 cells. (G) The level of CK-MB in in H9c2 cells. (H) SOD in H9c2 cells. (I) GSH-px in H9c2 cells. (J) MDS in H9c2 cells. (K) GSSG in H9c2 cells. (L) Basic ORC in H9c2 cells. (M) ORC in each group. Ctrl: Control; LPS; M-146a: LPS+M-exo-146a; M-IL-146a: LPS+M-IL-exo-146a; N-siRNA: LPS+*NF-κB1* siRNA; N-siRNA-M-146a: LPS+*NF-κB1* siRNA+M-exo-146a; N-siRNA-M-IL-146a: LPS+*NF-κB1* siRNA+M-IL-exo-146a. All the data was presented as mean±SD. Compared with control group: ^#^ *P*<0.05 and ^##^ *P*<0.01. Compared with LPS group: ^*^ *P*<0.05 and ^**^ *P*<0.01.

As shown in Figure 6A–C, LPS induced the release of TNF-α, IL-1β, and IL-6 in H9c2 cells, M-exo-146a and M-IL-exo-146a significantly decreased the release of TNF-α, IL-1β, and IL-6 of H9c2 cells. M-IL-exo-146a had a stronger effect than M-exo-146a and significantly decreased the levels of TNF-α, IL-1β, and IL-6. While, *NF-κB1* siRNA^−^ canceled the effects of M-exo-146a and M-IL-exo-146a [TNF-α: *F*(6, 35)=344.4, *P*<0.01; IL-1β: *F*(6, 35)=205.0, *P*<0.01; IL-6: *F*(6, 35)=264.6, *P*<0.01].

As shown in Figure 6D–E, the levels of ROS in H9c2 cell mitochondria, CK and CK-MB in H9c2 cells were significantly increased in LPS-treated group. M-exo-146a and M-IL-exo-146a significantly decreased the levels of ROS, CK, and CK-MB of H9C2 cells. M-IL-exo-146a had better effect than M-exo-146a. *NF-κB1* siRNA canceled the effects of M-exo-146a and IL-M-exo-146a [ROS: *F*(6, 35)=132.3, *P*<0.01; CK: *F*(6, 35)=118.9, *P*<0.01; CK-MB: *F*(6, 35)=239.5, *P*<0.01]. The level of MDA in H9c2 cell supernatant was increased, SOD and GSH-px were decreased compared to the control group, M-exo-146a and M-IL-exo-146a significantly reversed LPS-induced increase of MDA and decrease of SOD and GSH-px in cell supernatant. *NF-κB1* siRNA canceled the effects of M-exo-146a and M-IL-exo-146a (Fig. 6F–K) [MDA: *F*(6, 14)=53.04, *P*<0.01; SOD: *F*(6, 14)=65.23, *P*<0.01; GSH-px: *F*(6, 14)=43.64, *P*<0.01; GSSG: *F*(6, 14)=52.18, *P*<0.01].

### The effects of M-IL-exo-146a on mitochondrial function in H9c2 cells

Mitochondrial OCR was measured using Agilent Seahorse XF technology. As compared with the control group, LPS significantly reduced the OCR of H9c2 cells. M-exo-146a and M-IL-exo-146a significantly increased OCR, and M-IL-exo-146a had a better effect than M-exo-146a. *NF-κB1* siRNA canceled the effects of M-exo-146a and M-IL-exo-146a (Fig. 6L, M) [*F*(6, 77)=13.83, *P*<0.01].

### The effects of M-IL-exo-146a on protein expression of mitochondrial respiratory complex (CIII-core2, CII-30, and CIV-II) in H9c2 cells

**Figure 7:**
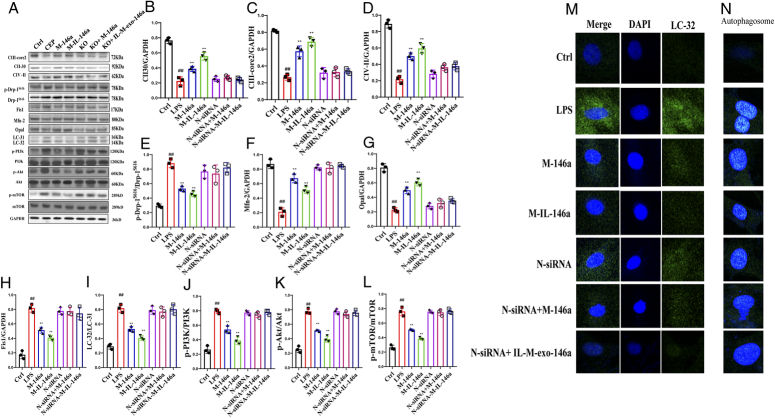
The effects of M-IL-exo-146 on mitochondrial respiration, fusion division, autophagy-related proteins protein, and autophagosome in H9c2 cells. (A) The levels of CIII-core2, CII-30 and CIV-II, p-Drp-1^S616^, Fis1, Mfn-2, Opal, LC-32/LC-31, p-PI3K, p-Akt, and p-mTOR were detected via Western Blot. (B) The quantification levels of CIII-core2. (C) The quantification level of CII-30. (D) The quantification level of CIV-II. (E) The quantification level of p-Drp-1^S616^. (F) The quantification level of Fis1. (G) The quantification level of Mfn-2. (H) The quantification level of Opal. (I) The quantification level of LC-32/LC-31. (J) The quantification level of p-PI3K. (K) The quantification level of p-Akt. (L) The quantification level of p-mTOR. (M) Autophagosome in H9c2 cells. Original magnification: 200, scale bar: 20 μm. Ctrl: Control; LPS; M-146a: LPS+M-exo-146a; M-IL-146a: LPS+M-IL-exo-146a; N-siRNA: LPS+*NF-κB1* siRNA; N-siRNA-M-146a: LPS+*NF-κB1* siRNA+M-exo-146a; N-siRNA-M-IL-146a: LPS+*NF-κB1* siRNA+M-IL-exo-146a. All the data was presented as mean±SD. Compared with the control group: ^#^ *P*<0.05 and ^##^ *P*<0.01. Compared with the LPS group: ^*^ *P*<0.05 and ^**^ *P*<0.01.

As shown in Figure 7A–L, compared with the control group, the levels of CIII-core2, CII-30, and CIV-II were significantly decreased in the LPS-treated group. M-exo-146a and M-IL-exo-146a significantly increased the levels of CIII-core2, CII-30, and CIV-II in H9c2 cells. M-IL-exo-146a significantly increased the levels of CIII-core2, CII-30, and CIV-II. *NF-κB1* siRNA canceled the effects of M-exo-146a and M-IL-exo-146a [CIII-core2: *F*(6, 14)=74.26, *P*<0.01; CII-30: *F*(6, 14)=60.82, *P*<0.01; CIV-II: *F*(6, 14)=71.33, *P*<0.01].

### The effect of M-IL-exo-146a on mitochondrial division and fusion proteins in H9c2 cells

As shown in Figure 7A–L, compared with the control group, the levels of p-Drp-1^S616^ and Fis1 were significantly increased, and Mfn-2 and Opal significantly decreased. M-exo-146a and IL-M-exo-146a markedly increased the levels of Mfn-2 and Opal and decreased p-Drp-1^S616^ and Fis1 in H9c2 cells. As compared with the M-exo-146a, M-IL-exo-146a had a better effect than M-exo-146a. *NF-κB1* siRNA canceled the effects of M-exo-146a and M-IL-exo-146a [Mfn-2: *F*(6, 14)=62.40, *P*<0.01; Opal: *F*(6, 14)=65.73, *P*<0.01; p-Drp-1^S616^/p-Drp-1^S616^: *F*(6, 14)=28.63, *P*<0.01; Fis1: *F*(6, 14)=55.74, *P*<0.01].

### The effects of M-IL-exo-146a on myocardial mitochondrial autophagy in H9c2 cells

As shown in Figure 7A–L, compared with the control group, levels of LC-32/LC-31, p-PI3K, p-Akt, and p-mTOR were increased in the LPS-treated group. M-exo-146a and M-IL-exo-146a significantly decreased levels of LC-32/LC-31, p-PI3K, p-Akt, and p-mTOR in H9c2 cells. M-IL-exo-146a had better effect on levels of LC-32/LC-31, p-PI3K, p-Akt, and p-mTOR in H9c2 cells. *NF-κB1* siRNA canceled the effects of M-exo-146a and M-IL-exo-146a. The immunofluorescence result of Nrf-2 was consistent with the above results (Fig. 7K). In order to further detect the effect of M-exo-146a and IL-M-exo-146a on autophagy, acridine orange staining was used to observe the effect of M-exo-146a and M-IL-exo-146a on autophagosome of H9c2 cells. As shown in Figure 7L, compared with the control group, the level of autophagosome was increased. M-exo-146a and M-IL-exo-146a significantly decreased the level of autophagosome in H9c2 cells. M-IL-exo-146a had a stronger effect than M-exo-146a. *NF-κB1* siRNA canceled the effects of M-exo-146a and M-IL-exo-146a [LC-32/LC-31: *F*(6, 14)=54.92, *P*<0.01; p-PI3K/PI3K: *F*(6, 14)=77.53, *P*<0.01; p-Akt/Akt: *F*(6, 14)=101.0, *P*<0.01; p-mTOR/mTOR: *F*(6, 14)=114.3, *P*<0.01].

### IL-1β-induced activation of the NF-κB pathway

**Figure 8:**
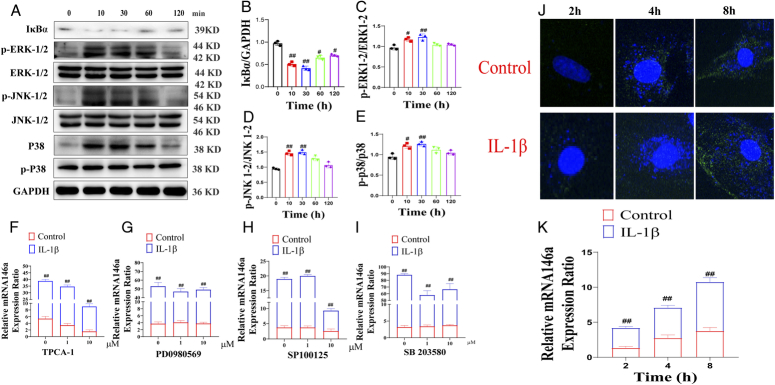
In-vitro validation of mechanism of IL-1β on miRNA146a. (A) The IκBa, p-ERK-1/2, p-JNK-1/2, and p-p38 protein expression at 0, 10, 30, and 60 min after IL-1β stimulation. (B) The quantification level of IκBa. (C) The quantification level of p-ERK-1/2. (D) The quantification level of p-JNK-1/2. (E) The quantification level of p-p38. (F) The levels of miRNA146a after TPCA-1 stimulation. (G) The levels of miRNA146a after PD0980569 stimulation. (H) The levels of miRNA146a after SP100125 stimulation. (I) The levels of miRNA146a after SB203580 stimulation. (J) The uptake of exo-mirna146a by H9c2 cells at 2, 4, and 8 h after IL-1β stimulation. (K) The levels of miRNA146a in H9c2 cells after IL-1β stimulation.

Before we detected the effects of TPCA-1, PD098059, SB600125, and SB203580 (IKK, ERK-1/2, JNK-1/2, and p38 MAPK inhibitor), we first detected whether the IKK signal was involved in the effect of IL-1β on miRNA146a. Results showed that activated IKK-2 could phosphorylate IκBa, resulting in the separation of IκBa from NF-κB, which is then degraded rapidly after ubiquitination. The activation of IKK-2 and NF-κB was indicated by measuring the total level of IκBa in LPS-treated H9C2 cells by Western blotting, which showed a rapid and transient loss of IκBa expression after IL-1B stimulation within 10 min and recovered after 60–120 min. Activation of the MAP kinase pathway was also confirmed by Western blotting using phosphorylated antibodies of ERK-1/2 (Thr202/Tyr204), JNK-1/2 (Thr183/Thr185), and p38 MAP kinase (Thr180/Try182). IL-1β induced rapid activation of ERK-1/2, JNK-1/2, and p38 MAP kinases, where phosphorylation levels were increased at 10 min and remained at the elevated level throughout 120 min (Fig. 8A–E) [IκBa/GAPDH: *F*(4, 10)=69.90, *P*<0.01; p-ERK-1/2/ERK-1/2: *F*(4, 10)=12.74, *P*<0.01; p-JNK-1/2/JNK-1/2: *F*(4, 10)=32.78, *P*<0.01; p-P38/P38: *F*(4, 10)=11.09, *P*<0.01].

#### The effects of IKK, ERK-1/2, JNK-1/2, and p38 MAPK inhibitors on IL-1β-induced miR-146 expression

To determine the regulation mechanism of IL-1β on miRNA146a, we used several inhibitors to study the expression of related signal proteins. In this experiment, we selected inhibitors TPCA-1 (IKK-2 inhibitor), PD098059 (ERK kinase-1/2 or MEK-1/2 inhibitor, ERK-1/2 upstream activator, SB600125 (JNK-1/2 inhibitor) and SB203580 (p38 MAP kinase inhibitor). Through PCR detection, the Δ CT of miRNA146a in LPS-treated cells was 15.38±2.5. After IL-1β stimulation, the Δ CT value of miRNA146a was increased 34.57 times. When pre-administration of IKK-2 inhibitor (TPCA-1), the level of miRNA146a was significantly decreased. Pre-administration of the MEK inhibitor PD98059 had no effect on the expression of mirRNA146a. After pre-administration of JNK-1/2 inhibitor sp600125, it was found that the expression of miRNA146a was significantly decreased. Pre-administration of p38 MAP kinase inhibitor SB203580 had no effect on the expression level of miRNA146a (Fig. 8F–I) [TPCA-1: *F*(2, 30)=678.2, *P*<0.01; PD098059: *F*(2, 30)=5.869, *P*<0.01; SB600125: *F*(2, 30)=203.7, *P*<0.01; SB203580: *F*(2, 30)=37.56, *P*<0.01].

#### Effect of IL-1β on exo-miRNA146a uptake by H9c2 cells

In the Transwell experiment, compared with the control group (there was no IL-1β in the upper chamber), immunofluorescence and PCR all showed that exo-miRNA146a was increased in H9c2 after IL-1β stimulation at 8 and 12 h (Fig. 8C, D) [*F*(2, 30)=16.55, *P*<0.01].

#### Detection results of exo-miRNA146a targeted *MAPK4* gene

**Figure 9:**
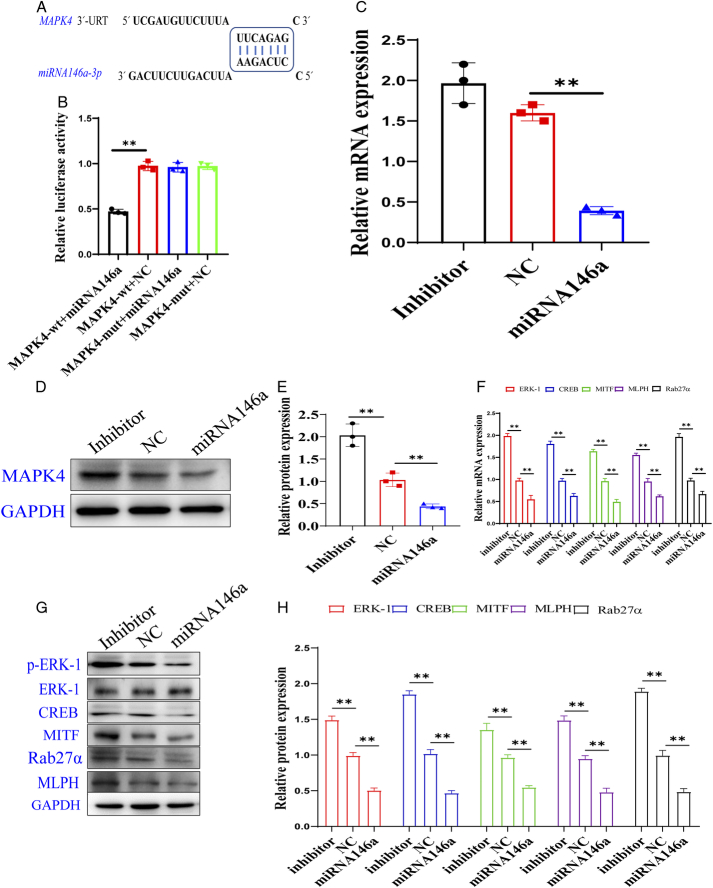
Detection results of miRNA146a targeted *MAPK4* gene (*n*=3). (A) Bioinformatics analysis using miRBase and Targetscan software, a potential target of miRNA146a was found in MAPK4 mRNA. (B) miR-146a expression level under different conditions. All the data was presented as mean±SD. Compared with *MAPK4*-wt+miRNA146a group: ^*^ *P*<0.05 and ^**^ *P*<0.01. (C) *MAPK*4 mRNA expression in H9c2 cells transfected with inhibitor, NC, and miRNA146a. (D) *MAPK*4 protein expression in H9c2 cells transfected with inhibitor, NC, and miRNA146a. (E) The quantification level of MAPK4 in H9c2 cells transfected with inhibitor, NC, and miRNA146a. All the data were presented as mean±SD. Compared with an inhibitor: ^*^ *P*<0.05 and ^**^ *P*<0.01. (F) *MAPK4*-related genes mRNA expression in H9c2 cells transfected with inhibitor, NC, and miRNA146a. All the data were presented as mean±SD. Compared with an inhibitor: ^*^ *P*<0.05 and ^**^ *P*<0.01. (G) *MAPK4*-related protein expression in H9c2 cells transfected with inhibitor, NC, and miRNA146a; (H) The quantification levels of MAPK4-related protein expression in H9c2 cells transfected with inhibitor, NC, and miRNA146a. All the data were presented as mean±SD. Compared with an inhibitor: ^*^ *P*<0.05 and ^**^ *P*<0.01.

Bioinformatics analysis showed the potential target of miRNA146a was MAPK4 mRNA. Compared with the wild-type 3′-URT, the double luciferase activity in the MAPK4-wt+miRNA146a group was significantly decreased, and the double luciferase activity in the mutant group did not significantly change (Fig. 9A, B) [*F*(3, 8)=100.1, *P*<0.01].

#### Effect of overexpression of miRNA146a on MAPK4 mRNA and protein expression

The effect of overexpression of miRNA146a on MAPK mRNA and protein was detected by PCR and WB. The results showed that after overexpression of miRNA146a, the *MAPK4* mRNA level in H9c2 cells significantly decreased compared with NC [*F*(2, 6)=80.39, *P*<0.01], and the MAPK4 protein expression level significantly decreased (Fig. 9C–E) [*F*(2, 6)=65.23, *P*<0.01].

#### Effect of overexpression of miRNA146a on MAPK4-related genes mRNA and protein

PCR and WB were used to detect the effect of miRNA146a on the expression of MAPK4-related genes and proteins. The results showed that after overexpression of miRNA146a, the ERK-1, CREB, MITF, ML-PH, and Rab27α mRNA [*F*(8, 30)=10.27, *P*<0.01] and protein expression [*F*(8, 30)=21.44, *P*<0.01] in H9c2 cells were significantly decreased compared with normal control (Fig. 9F–H).

### Verification of MAPK4 and Drp-1 interaction

#### MAPK4 binds to and phosphorylates Drp-1 *in vitro*

**Figure 10:**
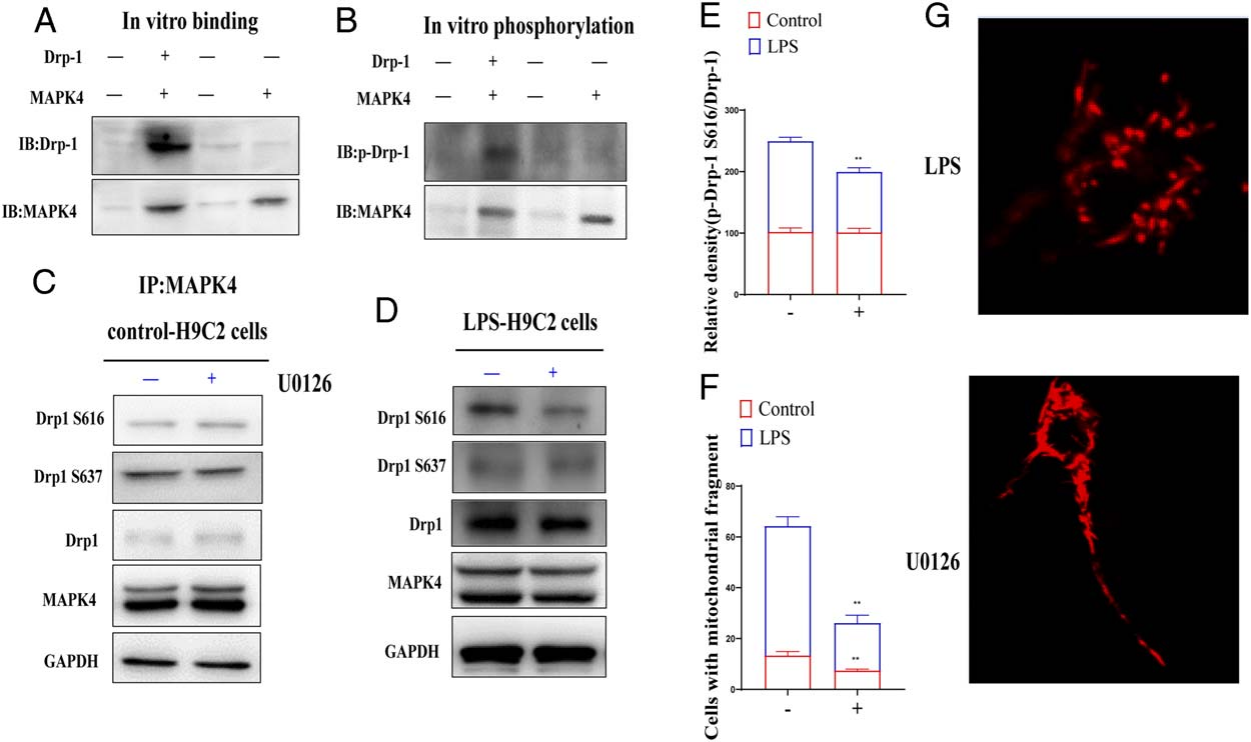
Verification of MAPK4 and Drp-1: MAPK4 binds to and phosphorylates Drp-1 *in vitro*. (A) Immunoprecipitation (IP) was performed with anti-MAPK4. (B) Western blotting with anti-serine/threonine antibody showed that Drp-1 was phosphorylated by MAPK4. (C) The level of p-Drp-1^S616^, p-Drp-1^S637^, Drp-1, and MAPK4 in U0126 (10 μM)-treated H9c2 cells were analyzed by Western Blot. (D) The quantification of p-Drp-1^S616;^ in U0126 (10 μM)-treated H9c2 cells and LPS-induced H9c2 cells; (E) The relative density of Drp-1 s616 relative to the total level of Drp-1. All the data were presented as mean±SD. Compared with control: ^*^ *P*<0.05 and ^**^ *P*<0.01. (G) U0126-treated H9c2 cells were stained with the mitochondrial protein FITC-Tom20. All the data were presented as mean±SD. Compared with control: ^*^ *P*<0.05 and ^**^ *P*<0.01.

To determine whether MAPK4 can phosphorylate Drp-1, recombinant human Drp-1 (GST-Drp-1) was phosphorylated *in vitro* in the presence of ATP. IP analysis showed that Drp-1 interacted with MAPK4. In addition, we found that Drp-1 was phosphorylated in the presence of MAPK4 when using antibodies that recognize phosphoserine/threonine (Fig. 10A, B).

#### MAPK4 induced increase of Drp-1 ser616 phosphorylation in H9c2 cells

To determine the phosphorylation of Drp-1 at specific serine sites, total lysates were collected from H9c2 cells and analyzed by WB using anti-p-Drp-1 s616, anti-p-Drp-1 s637, and anti-Drp-1 antibodies. We found that compared with normal H9c2 cells, LPS induced increased Drp-1 phosphorylation at ser616 in H9c2 cells, while ser637 phosphorylation did not change (Fig. 10C–E).

#### MAPK4 inactivation reduced mitochondrial fragmentation in H9c2 cells

In order to better explain the functional significance of MAPK4 phosphorylated Drp-1, the morphology of mitochondria in H9C2 cells was determined by immunostaining with an anti-Tom20 antibody (Tom 20, mitochondrial outer membrane protein). LPS-treated H9c2 cells showed excessive mitochondrial fragmentation, which was characterized by dot or globular by Tom20 positive staining. U0126 treatment improved the mitochondrial network. We observed elongated mitochondria and interconnected mitochondrial tubular networks. These results suggested that MAPK4 played an important role in the regulation of mitochondrial morphology in H9c2 cells (Fig. 10F, G).

## Discussion

At present, the efficacy of traditional drugs for treating SMI is still not optimistic, and there is no fully effective drug for SMI treatment in clinical practice. In recent years, a large number of basic studies have suggested that certain phytochemical or bioactive drugs exhibit certain SMI therapeutic effects^12^. However, the effectiveness of drug therapy was limited^13^. In recent years, some scholars have started to use cytokines, LncRNAs, miRNAs, and other methods to treat SMI, but due to the existence of some unstable factors, the effect is not satisfactory. This study utilized IL-1β-stimulated macrophage-derived exosomes miRNA146a for the treatment of SMI, and elucidating their specific mechanisms of action provided a potential novel treatment approach for SMI.

Exosomes have been shown to perform many pharmacological activities. Specifically, exosomes have exhibited inspiring anti-inflammatory activities. Exo has been reported to treat heart disease. Adipose tissue macrophage-derived exosomes significantly alleviated obesity-induced cardiac injury^14^. There is a certain coincidence with previous reports; we found a potent ability of M-IL-exo-146a to alleviate SMI. Furthermore, M-IL-exo-146a was found to have an anti-inflammation effect and mitochondrial function improvement. The mechanism that IL-1β enhances the protective effect of M-exo on SMI is due to increasing the expression of miR-146a in M-exo. These findings have major implications for the potential treatment of M-IL-exo-146a on SMI.

Oxidative stress plays an important role in SMI. LPS induces excessive production of ROS, and ROS accumulation triggers inflammation or cell death, which has been implicated in the pathogenesis of sepsis. Under physiological conditions, ROS production is counteracted by various cellular antioxidant enzymes to promote redox homeostasis^15^. However, ROS accumulation can lead to an imbalance of oxidants and antioxidant capacity. MDA, a marker of lipid peroxidation and antioxidant enzymes, including SOD and GSH-Px, reflects the extent of oxidative stress. Our study showed that sepsis increased MDA levels and decreased SOD and GSH-Px levels in the heart tissue of mice, indicating that sepsis can increase the generation of ROS. M-exo-146a and M-IL-exo-146a significantly decreased the generation of ROS. Furthermore, M-IL-exo-146a had a better effect than M-exo-146a. It suggests that IL-1β has an enhancing effect on M-exo-146a.

Inflammatory cytokines such as IL-1, IL-6, IL-18, and TNF-α are involved in the pathogenesis of sepsis^16^. IL-1β is synthesized by a variety of cell types, including macrophages, monocytes, and fibroblasts, and is an effective mediator of inflammation and immunity. In many sepsis animal models, IL-1β was increased^17^. IL-6 is another important inflammatory cytokine, and IL-6 mRNA was demonstrated that in the lung with sepsis, and serum IL-6 is a candidate marker for sepsis^18^. TNF-α is a pro-inflammatory cytokine secreted from monocytes and macrophages that is functional in lipid metabolism, insulin resistance, and endothelial biology. TNF-α involved in the pathogenesis of sepsis, and inhibition of it would have significant therapeutic effects^19^. Our present study found that M-exo-146a and IL-M-exo-146a could significantly decrease the release of cytokine of macrophage *in vitro* and *in vivo*. Knock out or silence NF-κB could antagonize the effect of M-exo-146a and IL-M-exo-146a on the release of cytokine. It is suggested that miR-146a antagonizing inflammatory response is mainly related to the NF-κB signal pathway.

Myocardial injury in sepsis cannot be explained solely by insufficient perfusion of tissues and organs, in which mitochondrial and non-mitochondrial mechanisms (including hemodynamic changes, myocardial inhibitory factors, inflammatory mechanisms, immune regulation mechanisms, etc.) play an important role. The mitochondrial mechanism is extremely complex, and it is a key participant in the pathophysiology of SMI. The abnormalities of its structure, quantity, and function play a very important role in SMI. Therefore, the targeted therapy of myocardial mitochondria in sepsis came into being^20^. Mitochondrial structure damage in SMI is usually manifested as myocardial mitochondria DNA damage, matrix edema, mitochondrial inner and outer membrane damage, and internal vesicle formation, accompanied by ridge loss or destruction and so on. Avoiding mitochondrial structural damage is very important to ensure the normal function of mitochondria. Our present study found that M-exo-146a and IL-M-exo-146a could significantly improve myocardial mitochondrial function and mitochondrial homeostasis. Mitochondria are the key organelles of the cell, which produce ATP through aerobic metabolism to provide energy for cells. As a by-product of oxidative phosphorylation, mitochondria continue to produce cellular ROS. During cell stress, the level of mtROS will significantly increase. After entering the cytoplasm, it can lead to mitochondrial imbalance^21–23^. In this study, we found that LPS could significantly cause mitochondrial homeostasis imbalance, and M-exo-146a and M-IL-exo-146a significantly improve myocardial mitochondrial imbalance in sepsis. It is suggested that M-IL-exo-146a alleviates SMI, except for anti-oxidative stress injury and anti-inflammatory response, and is also related to the improvement of mitochondrial function.

How does miRNA146a regulate mitochondrial homeostasis? Is the key issue of this study. First, we found that there was a potential target of miRNA146a in MAPK4 miRNA by the bioinformatics method. Compared with the wild-type 3′-URT, the double luciferase activity of the MAPK4-wt+miRNA146a group significantly decreased. These results suggested that the MAPK4 gene was the target gene of miRNA146.

Explaining how MAPK4 regulates mitochondrial function will be the next step. Drp-1 is a cytoplasmic GTPase that regulates mitotic events. It can be modified after translation, such as phosphorylation, so as to change the activity and location of proteins. Ser637 of Drp-1 is phosphorylated by protein kinase A (PKA), which reduces its GTPase activity and protects mitochondria. During oxidative stress, protein kinase Cδ (PKC-δ) phosphorylation of ser616 of Drp-1 leads to mitochondrial disruption and dysfunction. This study found that MAPK4 phosphorylated Drp-1 at ser616. Increased ser616 phosphorylation was associated with increased mitotic division. Excessive mitosis of mitochondria leads to mitochondrial rupture, which leads to mitochondrial outer membrane permeability, ATP depletion, ROS increase and apoptotic factor release. At the same time, it was found that the inactivation or inhibition of MAPK4 would stabilize the function of mitochondria and reduce mitochondrial fragmentation, which was due to the reduction of Drp-1 phosphorylation.

Why IL-1β can enhance the protection of M-exo on SMI, we observed the effect of IL-1β on the expression of miR-146a in M-exo and its relationship to ERK, JNK, and MAPK pathway. The results showed that IKK-2 inhibitor, TPCA-1 and JNK-1/2 inhibitor sp600125 significantly inhibited the effect of IL-1β on miRNA146a. While ERK-1/2 inhibitor PD98059 and p38 MAP kinase inhibitor SB203580 had no effect on the effect of IL-1β on miRNA146a. It is suggested that IL-1β enhancing the expression of miRNA146a may be related to JNK-1/2 pathway.

## Conclusions

**Figure 11:**
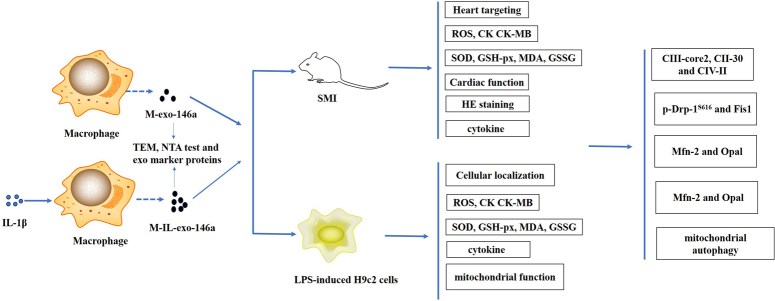
The scheme pathway of M-IL-exo-146 on SMI *in vitro* and *in vivo* in this study.

To sum up, M-exo-146a and M-IL-exo-146a have a good protective effect on SMI *in vitro* and *in vivo* (Fig. 11). IL-M-exo-146a has a better effect than M-exo-146a. IL-1β enhancing the effect of M-exo-146a was due to increasing the expression of miR-146a in M-exo and IL-M-exo-146a alleviating SMI was related to anti-oxidative stress and anti-inflammation, and mitochondrial function improvement and this finding provided a method to treat SMI.

### Limitations

Although this study explains the role and mechanism of M-IL-exo-146a on SMI, there are two limitations: (1) This study was only limited to the mouse level and does not involve big animals and humans. (2) this study primarily investigated the mechanism of IL-1β enhancing the effect of M-exo-146a, but the in-depth mechanism was not described. (3) At present, research is only limited to a small number of animals, requiring a large number of animals and large animals such as monkeys for research. (4) The amount and quantity of exosomes in this study were small, and it was also necessary to design experiments on how to generate large quantities and quantities of exosomes, which was also one of the shortcomings of this study.

### Translational aspects

This study has certain potential value for clinical translation. IL-M-exo-146a could serve as a new treatment for SMI in clinical practice and provide clinical physicians with a treatment approach that is currently considered effective. However, before clinical treatment could be applied, it was necessary to address the issues of exo contents and allogeneic exclusion reactions, which still required a large amount of preclinical experimental research.

## Ethical approval

All animals’ operations were approved by the Research Council and Animal Care and Use Committee of the Animal Centre of Army Medical University (application number 20221683). All patients authorized family members to sign informed consent, and the hospital approved the study of the Army Medical University ethics committee (KY20210756).

## Source of funding

Not applicable.

## Author contribution

C.M., J.W., Z.Y., and S.H.: did the experiments; L.T.: collected data and data analysis; X.M., T.L. and L.L.: designed experiments and wrote the paper.

## Conflicts of interest disclosure

The authors declare that they have no conflicts of interest.

## Research registration unique identifying number (UIN)

Not applicable.

## Guarantor

Chunhua Ma.

## Data availability statement

The data underlying this article are available on reasonable request to the corresponding author.

## Supplementary Material
