# Supplementary material for: Interleukin-1β-stimulated macrophage-derived exosomes improve myocardial injury in sepsis via regulation of mitochondrial homeostasis: experimental research
Source: Int J Surg. 2024 Jul 5;111(1):283–301. doi: 10.1097/JS9.0000000000001915 (PMC11745623; doi:10.1097/JS9.0000000000001915)
Supplement: Supplementary file 2 [file js9-111-0283-s002.docx]

**Methods**

**Different cytokines stimulate macrophages (M)**

M were seeded at 6-well-plate at a density of 1×10^5^ cells/well for 24 h. M were divided into four groups: control group (Ctr), interleukin-6(IL-6, 10 ng/mL), tumor necrosis factor-α (TNF-α, 10 ng/mL), IL-10 (10ng/mL) and IL-1β stimulation groups. The cells of IL-6, TNF-α, IL-10 and IL-1β stimulation groups were treated IL-6 (10 ng/mL), TNF-α (10 ng/mL), IL-10 (10 ng/mL) and IL-1β (10 ng/mL) for 24h, while the control group was challenged with an equal PBS.

**Extraction of** **different cytokines stimulate M-derived exosomes**

Exo-Quick Precipitation method was used to extract different cytokines stimulate M-derived exosomes (IL-6-exo, IL-1β-exo, TNF-α-exo and IL-10-exo), and the specific method was strictly in accordance with the instructions of the kit.

**Protein quantification of different cytokines stimulate M-derived exosomes**

1/4 volume of RIPA protein lysate (RIPA protein buffer) was added into different cytokines stimulate M-derived exosomes, mixed evenly, placed in an ice box, and incubated for 30 min until exosomes were completely lysed. Then, the contents of proteins were detected according to BCA kit instructions.

**In vitro model establishment**

H9c2 cells were seeded at 6-well-plate at a density of 1×10^6^ cells/well for 24 h. After that, the cells were divided into these groups: control, LPS model (50 µM), LPS + IL-6-exo (10 µg/mL), LPS+ IL-1β-exo (10 µg/mL), LPS + TNF-α-exo (10 µg/mL), LPS + IL-10-exo (10 µg/mL). The cells were treated with for IL-6-exo, IL-1β-exo, TNF-α-exo and IL-10-exo 24h, and 50 µM LPS were co-incubated for 24h, while the control group was challenged with an equal PBS. Cell supernatant and cells were collected for subsequent detection.

**Cell viability**

H9c2 cells were seeded at 6-well-plate at a density of 1×10^6^ cells/well for 24 h. After that, the cells were divided into these groups: control, LPS model (50 µM), LPS + IL-6-exo (10 µg/mL), LPS+ IL-1β-exo (10 µg/mL), LPS + TNF-α-exo (10 µg/mL), LPS + IL-10-exo (10 µg/mL). The cells were treated with for IL-6-exo, IL-1β-exo, TNF-α-exo and IL-10-exo 24h, and 50 µM LPS were co-incubated for 24h and the CCK-8 kit was used to detect cell viability.

**Measurement of myocardial isozymes (CK-MB)**

At the end of the experiment, the level of CK-MB of H9c2 cell supernatant detected by commercial kits. The operation process strictly followed the instructions of the kits.

**Results**

**The results of protein quantification of** **different cytokines stimulate M-derived exosomes**

The protein concentrations of total protein of IL-6-exo, IL-1β-exo, TNF-α-exo and IL-10-exo (IL-M-exo) were 17.49 mg/mL, 18.56 mg/mL, 17.82 mg/mL and 18.31 mg/mL, respectively. This result shown the protein concentration of IL-1β-exo was the highest among them.

**
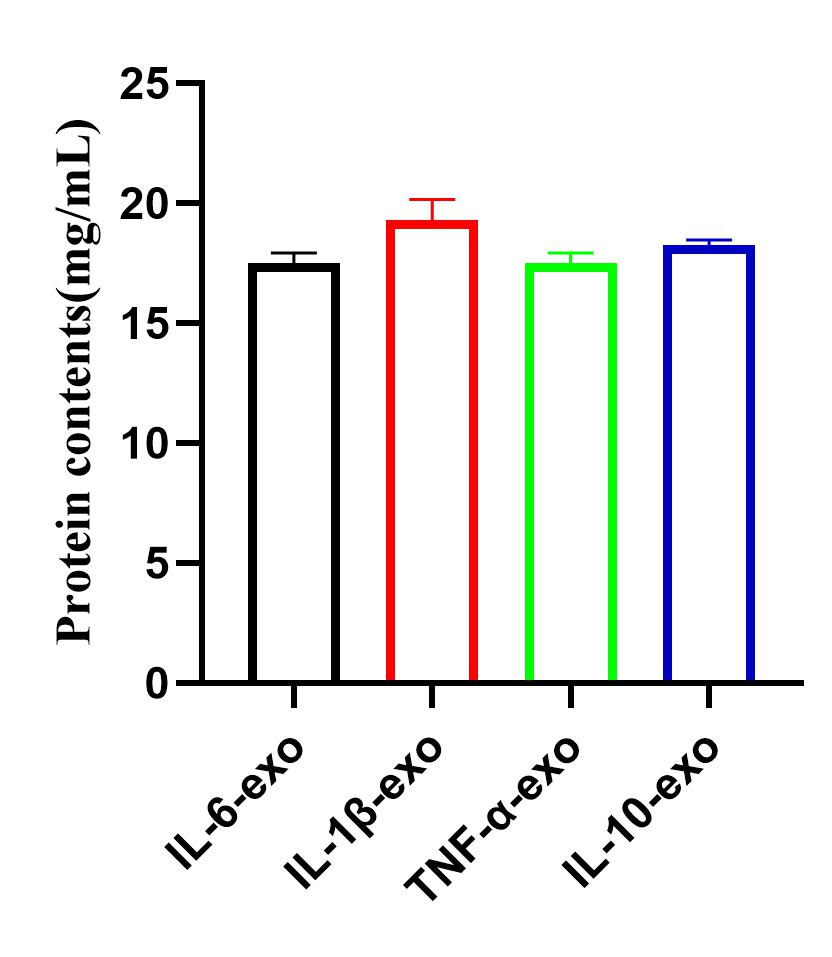
**

**The effects of different cytokines stimulate M-derived exosomes**

**on cell viability**

Compared with the control group, LPS significantly reduced cell viability, compared with LPS group, IL-6-exo, IL-1β-exo, TNF-α-exo and IL-10-exo increased cell viability in different degrees, and IL-1β-exo had the most obvious effect.

**
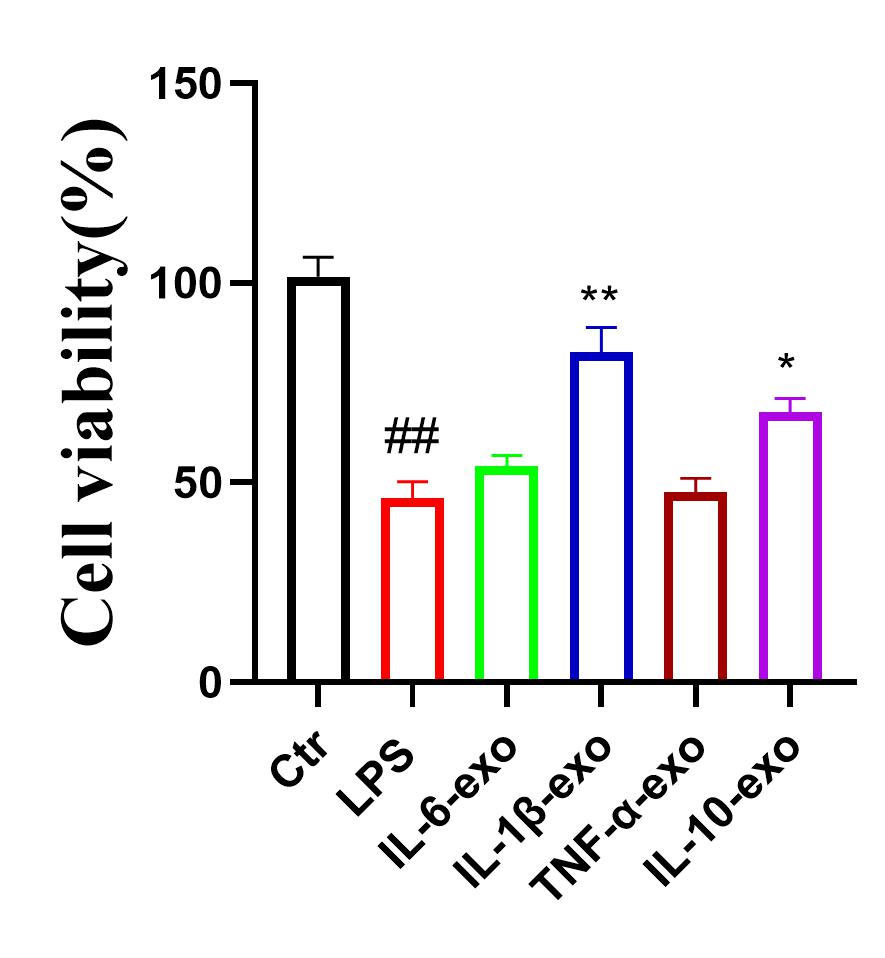
**

All the data was presented as mean ± SD. Compared with control group: ^#^P<0.05, ^##^P<0.01. Compared with LPS group: ^*^P<0.05, ^**^P<0.01.

**The effects of different cytokines stimulate M-derived exosomes**

**on CK-MB**

Compared with the control group, LPS significantly increased the level of CK-MB, compared with LPS group, IL-6-exo, IL-1β-exo, and IL-10-exo decreased CK-MB in different degrees, TNF-α-exo has no effect of CK-MB and IL-1β-exo had the most obvious effect.
